# Postinfectious Onset of Myasthenia Gravis in a COVID-19 Patient

**DOI:** 10.3389/fneur.2020.576153

**Published:** 2020-10-06

**Authors:** Meret Huber, Sophia Rogozinski, Wolfram Puppe, Carsten Framme, Günter Höglinger, Karsten Hufendiek, Florian Wegner

**Affiliations:** ^1^Department of Neurology, Hannover Medical School, Hanover, Germany; ^2^Institute of Virology, Hannover Medical School, Hanover, Germany; ^3^Department of Ophthalmology, Hannover Medical School, Hanover, Germany

**Keywords:** anosmia/ageusia, COVID-19, diplopia, neurological manifestation, postinfectious myasthenia gravis, SARS-CoV-2

## Abstract

**Objective:** We report the case of a young woman with postinfectious onset of myasthenia gravis after COVID-19 with mild respiratory symptoms and anosmia/ageusia 1 month before admission to our neurological department.

**Methods:** Patient data were derived from medical records of Hannover Medical School, Germany. Written informed consent was obtained from the patient.

**Results:** The 21-year-old female patient presented with subacute, vertically shifted double vision evoked by right sided partial oculomotor paresis and ptosis. About 4 weeks earlier she had suffered from mild respiratory symptoms, aching limbs and head without fever, accompanied by anosmia/ageusia. During the persistence of the latter symptoms for around 10 days the patient had already noticed “tired eyes” and fluctuating double vision. Clinical assessment including a positive test with edrophonium chloride and increased acetylcholine receptor antibodies related the ocular manifestation etiologically to myasthenia gravis. Antibodies (IgA/IgG) against SARS-CoV-2 using three different serological tests (Abbott, DiaSorin, Euroimmun) were detected in serum suggesting this specific coronavirus as previously infectious agent in our patient. The myasthenic syndrome was treated successfully with intravenous immunoglobulins and oral pyridostigmine.

**Conclusion:** This is the first case presentation of postinfectious myasthenia gravis as neurological complication in a COVID-19 patient.

## Introduction

Besides respiratory symptoms of COVID-19, frequent neurological manifestations including prolonged anosmia/ageusia during the acute SARS-CoV-2 infection have been described (1). Additionally, there are few case reports on postinfectious neurological diseases like the Guillain-Barré-Syndrome (2–4) and Miller-Fisher-Syndrome (5).

We present the first case of myasthenia gravis as neurological complication after SARS-CoV-2 infection.

## Methods

Euroimmun (Lübeck, Germany) provided SARS-CoV-2 ELISA IgA/IgG and PCR. Two additional ELISA IgG tests from Abbott (Sligo, Ireland) and DiaSorin (Sasluggia, Italy) were used to validate serological results.

Written informed consents were obtained from the patient (consent for publication and consent to disclose photographs/video).

## Case Report

A 21-year-old woman was admitted via the emergency room at Hanover Medical School in mid-April 2020 because of double vision and right-sided ptosis which had progressively worsened for 5 days. The patient stated that by end of March she and her family members had suffered from mild respiratory symptoms without fever, moderate fatigue with aching limbs and head, dry eyes and nasal mucosa as well as anosmia/ageusia. Until April all affected family members recovered without having been tested for SARS-CoV-2 using PCR or antibody assays.

The patient's medical history had been been unremarkable so far. While there is no family history of neuromuscular disorders at all, there are cases of Hashimoto's thyreoiditis, Addison's disease and pernicious anemia in her family. However, the three siblings of the patient are healthy, she has no children.

**Figure 1:**
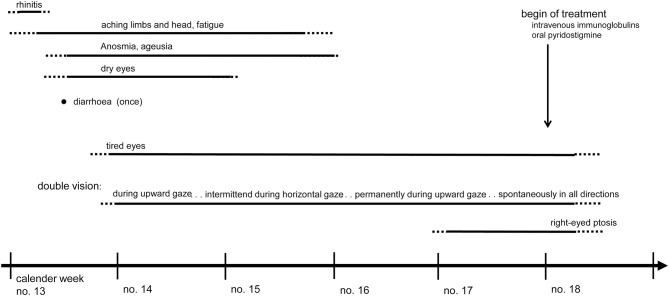
Time line of COVID-19 symptoms and overlapping onset of ocular myasthenia gravis which was treated successfully with intravenous immunoglobulins and oral pyridostigmine.

Most of the patient's COVID-19 related symptoms had regressed within 3 weeks when ocular symptoms evolved post-infectiously (Figure 1). The patient experienced initially unspecific visual symptoms (“tired eyes”) until intermittent but overall progressive double vision lead to admission.

Neuro-ophthalmological examination in mid-April showed a right eyed elevation deficit as cause of vertical double images and ptosis. An MRI scan with contrast enhancement displayed regular anatomical structures of brain and orbit. An oropharyngeal swab taken about 4 weeks after onset of respiratory symptoms was tested negative by PCR indicating no acute SARS-CoV-2 infection (6).

Two days later, neurological examination revealed increasing oculomotor impairment due to right eyed elevation deficit and ptosis with positive Cogan lid twitch test (Besinger score 7). During investigation the patient described vertically offset double vision in all directions with reduced double images when looking to the left. The remaining neurological status was normal.

**Figure 2:**
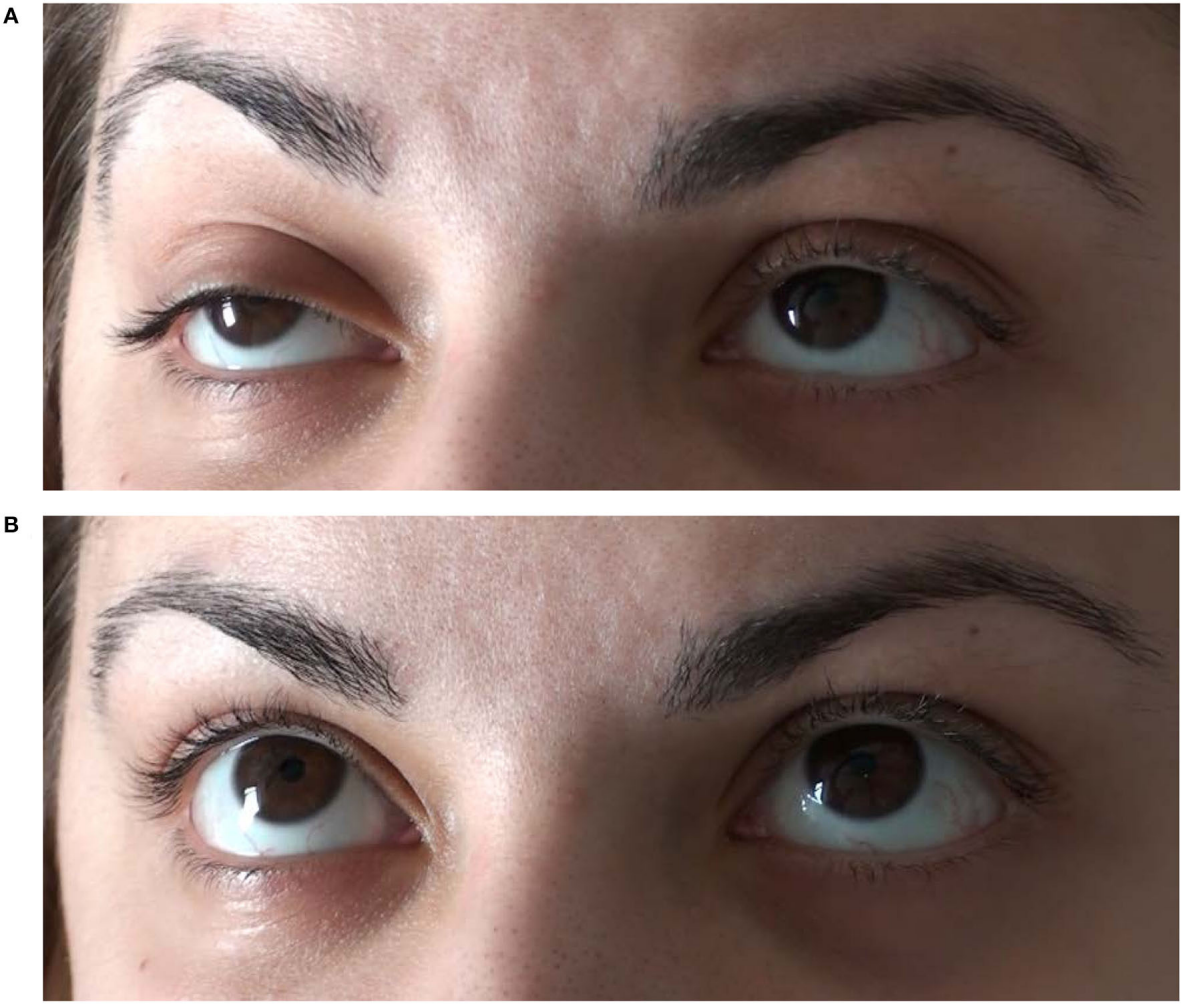
Mainly right sided oculomotor paresis with elevation deficit and ptosis **(A)** responded positively to an intravenous test dose of 9 mg edrophonium chloride **(B)** suggesting that double vision was caused by ocular manifestation of myasthenia gravis.

Electrophysiological examinations showed inconspicuous motor and sensory neurographies without increment. In repetitive stimulations no decrement (3 Hz) of the orbicularis oculi or trapezius muscles could be detected. The Simpson test showed a significant worsening of ptosis and double vision during forced upward gaze after 20 s. An ice on eyes test was negative, whereas a test with intravenous edrophonium chloride (9 mg cumulative dose) was clearly positive (full recovery of forced upward gaze for 45 s; Figure 2).

Antibodies against acetylcholine receptors were elevated in serum (0.9 nmol/l instead of <0.25 nmol/l). Further blood investigations including other myasthenia-associated antibodies (Agrin, LRP4, MuSK, Titin) and screening parameters for immunological diseases including ganglioside IgM/IgG remained unremarkable. SARS-CoV-2 IgA and IgG ELISA (Euroimmun) were positive in serum underlining the typical history of COVID-19 in our patient. Two additional IgG ELISA tests (Abbott, DiaSorin) were performed to validate the positive SARS-CoV-2 serological results, an IgM test was not available. The microscopical CSF cell count, protein and lactate were normal, oligoclonal bands positive (type 2a). The PCR and IgG/IgA ELISA for SARS-CoV-2 in the CSF were negative (Euroimmun, performed experimentally). Further examinations of serum and CSF on herpes infection, borreliosis and lues showed no indication for acute infection.

In the absence of dysphagia and dyspnea, standardized lung function tests remained inconspicuous. Electroencephalographical findings were unremarkable. Further examinations with thoracic X-ray and MRI did not show a thymoma. Lung parenchyma displayed no (post)inflammatory changes.

Due to acute clinical exacerbation over the last days, the patient was treated with intravenous immunoglobulins (7) (0.4 g/kg/d for 5 days) and oral pyridostigmine (3 × 60 mg/d*)* resulting in regression of ocular symptoms (Besinger score 3) before discharge. After gradual increase of oral pyridostigmine up to 3 × 120 mg/d during the next weeks further improvement was noted at a follow up in mid-May (Besinger score 0).

## Discussion

In this case report we present a young woman with subacute ocular manifestation of myasthenia gravis after a typical COVID-19 infection including neurological symptoms (headache, anosmia/ageusia). Anosmia/ageusia are commonly reported neurological symptoms of SARS-CoV-2 infections (8). Most COVID-19 patients do not suffer from nasal obstruction or rhinitis symptoms, so possibly anosmia/ageusia are neurological complications due to direct damage to the receptors (8).

Myasthenia gravis is an autoimmune, neuromuscular disease that is mostly caused by antibodies against postsynaptic acetylcholine receptors. Generally, there are only few recent reports about post-infectious myasthenia gravis [after varicella-infections (9, 10)/viral pharyngitis (10)]. One report about an acute general flaccid paralysis in a child without CSF abnormalities was related to a coronavirus-infection with HCoV 229E and OC43 (11). In contrast to previous cases of post-viral myasthenia gravis (9, 10), we detected elevated acetylcholine receptor antibodies in our patient. It remains speculation whether a potential molecular mimicry between the acetylcholine receptor and SARS-CoV-2 proteins might have triggered the immune response leading to post-infectious onset of myasthenia gravis (12). However, there seems to be no marked structural identity between the relevant nicotinic acetylcholine receptor subunits (alpha1, beta1, delta, epsilon) and known SARS-CoV-2 proteins (https://www.uniprot.org/blast).

In our patient's serum, IgA and IgG antibodies directed against SARS-CoV-2 were detected in calendar week 17, presumably 4 weeks after initial respiratory symptoms. We used a semiquantitative ELISA IgA/IgG test which detects the S1 domain reaching a specificity of 98.5% (for IgG)/92.5% (for IgA, both provided by Euroimmun). Validation of serological results was performed using two different ELISA IgG tests with a specificity of 98.5% (DiaSorin) and 99.45% (Abbott) applied >14 days after infection. However, cross-reactions to cytomegalovirus IgG (Abbott), hepatitis B and influenza A (DiaSorin) or other coronaviruses, especially SARS-CoV-1 (Euroimmun), are known to manufacturers. Due to typical COVID-19 symptoms in our patient's history and the multiple positive serological test results for SARS-CoV-2 we highly assume a preceding infection with this new coronavirus triggering post-infectious ocular manifestation of myasthenia gravis as neurological complication.

On the other hand a COVID-19 patient might incidentally suffer from overlapping onset of myasthenia gravis. In our opinion this issue cannot be clarified completely even by retrospective comparison of disease occurrence rates. Despite the occurrence rate of SARS-CoV-2 infections in our area at the end of March 2020 was moderately high (7-day incidence of 20–28 new infections/100,000 citizens in Lower Saxony according to the Robert-Koch-Institute) and myasthenia gravis is considered a rare disease (incidence of 0.25–2/100,000 citizens according to the guidelines of the German Society of Neurology) we cannot rule out that myasthenia gravis might have been induced independently from the SARS-CoV-2 infection.

Although our patient does not have any preexisting illnesses and particularly no neuromuscular disorders, the family history is positive for autoimmune diseases. This suggests that COVID-19 patients with a similar history may be prone to suffer from post-infectious neuroimmunological complications due to SARS-CoV-2.

## Conclusion

We present the first case of post-infectious myasthenia gravis associated with SARS-CoV-2. An increasing number of patients with various neurological symptoms during acute infection or post-infectious complications have been reported but the full clinical spectrum and incidence of COVID-19 related neurological manifestations is still unknown. Future studies should address if there is an association higher than expected by chance.

## Data Availability Statement

All datasets generated for this study are included in the article/Supplementary Material.

## Ethics Statement

Ethical review and approval was not required for the study on human participants in accordance with the local legislation and institutional requirements. The patients/participants provided their written informed consent to participate in this study. Written informed consent was obtained from the individual(s) for the publication of any potentially identifiable images or data included in this article.

## Author Contributions

MH and FW: conception, organization and execution of the research project, writing of the first draft, and the review and critique of the manuscript. SR, WP, CF, GH, and KH: conception, organization and execution of the research project, review and critique of the manuscript.

## Conflict of Interest

The authors declare that the research was conducted in the absence of any commercial or financial relationships that could be construed as a potential conflict of interest.
